# Measles Outbreak Driven by Nosocomial Transmission, Armenia, February–July 2023

**DOI:** 10.3201/eid3202.250474

**Published:** 2026-02

**Authors:** Karo Palayan, Ani Manukyan, Gayane Sahakyan, Svetlana Grigoryan, Lilit Karapetyan, Shushan Sargsyan, Artavazd Vanyan, Pawel Stefanoff

**Affiliations:** Author affiliations: National Centre for Disease Control and Prevention, Ministry of Health, Yerevan, Armenia (K. Palayan, A. Manukyan, G. Sahakyan, S. Grigoryan, L. Karapetyan, S. Sargsyan, A. Vanyan); European Centre for Disease Prevention and Control, Mediterranean and Black Sea Programme in Intervention Epidemiology Training, Stockholm, Sweden (K. Palayan, P. Stefanoff)

**Keywords:** Measles, viruses, disease outbreaks, healthcare workers, Armenia

## Abstract

During March–July 2023, we investigated a measles outbreak in Armenia. Of 287 patients, 130 were <5 years of age and 215 (75%) were unvaccinated. Among 3 transmission chains involving 183 cases, 70% of patients were exposed in healthcare facilities. To minimize nosocomial transmission, measles vaccination should be encouraged among healthcare workers.

In 2002, Armenia adopted the World Health Organization (WHO)’s measles elimination plan and incorporated the trivalent live measles, mumps, and rubella (MMR) vaccine into its national immunization schedule. For children, the first dose (MMR1) was administered at 12 months of age, and the second dose (MMR2) at 6 years of age. In 2020, the recommended age for MMR2 was revised to 4–6 years. In 2007, after a large measles outbreak during 2004–2005 involving 4,064 cases, the Ministry of Health (MoH) implemented a supplementary immunization campaign targeting 1.2 million persons 6–27 years of age, offering 1 dose to each person (*1*,*2*).

During 2008–2022, Armenia reported primarily imported measles cases, and incidence rates remained <1 case/100,000 population. During 2009–2019, national 2-dose MMR vaccine coverage among target groups consistently exceeded the WHO’s 95% target. However, after 2020, coverage declined below 95% at national and regional levels (*3*,*4*).

On March 3, 2023, the Armenian National Center for Disease Control and Prevention (NCDC) received a report of 13 persons with suspected measles cases hospitalized at a 105-bed multidisciplinary pediatric hospital in Yereven. In response, NCDC established an outbreak investigation team to assess the extent of the outbreak and trace transmission chains to inform the national measles elimination strategy. We report findings from that activity, which was conducted as part of an emergency response and, in accordance with guidance from the Armenia MoH, did not require ethics approval.

## The Investigation

We defined a suspected case as illness in any person with clinical signs and symptoms, including fever and maculopapular rash, accompanied by cough, conjunctivitis, or both. We defined a confirmed case as a suspected case with laboratory confirmation of measles-specific IgM, an epidemiologic link to a laboratory-confirmed case, or both. During March–July 2023, local and regional outbreak investigation teams assessed each suspected case by using a standardized form that captured demographic information, clinical symptoms, and vaccination status. Local epidemiologists attempted to collect a serum specimen from each suspected case-patient, preferably 4–28 days after symptom onset. Upon laboratory confirmation, the investigation team contacted each confirmed case-patient to obtain a list of close contacts, defined as persons who shared a household, worked or were hospitalized in the same room, or frequently visited the same locations and for whom potential exposure occurred 6–18 days before rash onset.

Next, investigators interviewed each close contact for symptoms and vaccination status, informed contacts of their possible exposure, and offered MMR vaccination. Interviewers referred all close contacts who met the criteria for a suspected case to local epidemiologists for further investigation. Vaccination status was verified by using immunization records maintained at public health centers run by the MoH. We classified persons with unknown vaccination status as unvaccinated. The NCDC Reference Laboratory detected measles-specific IgM antibodies in serum specimens by using Measles IgM ELISA kits (EUROIMMUN, https://www.euroimmun.com), following WHO guidelines (*5*).

We defined a measles transmission chain as an uninterrupted series of epidemiologically linked confirmed cases, in which each link was supported by plausible close contact and rash onset dates separated by a 6–18-day incubation interval. We considered cases not linked if we could not identify an epidemiologic connection to other cases. We classified a confirmed case as imported if the person had traveled abroad during the incubation period and had no known close contact with a confirmed case.

During February 3–July 3, 2023, we interviewed ≈7,000 contacts, of whom 868 met the criteria for suspected measles cases. Among those, 284 were laboratory-confirmed and 3 were epidemiologically linked, resulting in a total of 287 confirmed cases. The epidemic curve indicated continuous measles transmission throughout the observation period (Figure). An additional 828 suspected cases were reported during August 2023–December 2024, an average of ≈49 cases per month, suggesting ongoing transmission.

**Figure:**
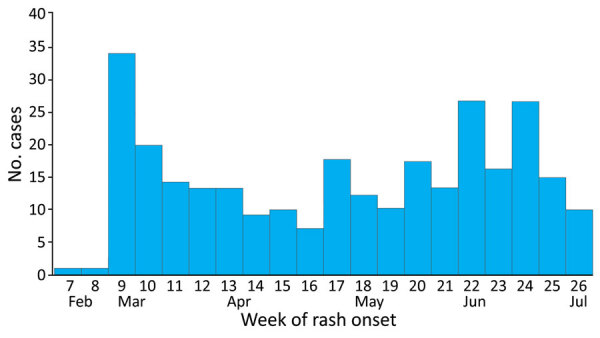
Number of confirmed cases by week of rash onset during measles outbreak driven by nosocomial transmission, Armenia, February–July 2023.

Of the 287 confirmed cases, 130 (45%) were among children <5 years of age, 152 (53%) case-patients were female and 135 (47%) were male, and 172 (60%) resided in Yerevan, the capital city. The median age was 8 (interquartile range [IQR] 1–25; range 0–64) years; 38 cases were imported. Healthcare workers (HCWs) accounted for 15 cases, of whom only 1 had received a single dose of a measles-containing vaccine. Among all 287 case-patients, 215 (75%) were unvaccinated, 29 (10%) had received 1 dose, 22 (7.7%) had received 2 doses, and 20 (7%) had unknown vaccination status (Table 1).

**Table 1:** Distribution of cases by age group and vaccination status during measles outbreak driven by nosocomial transmission, Armenia, February–July 2023.

Age range, y	Not vaccinated	1 dose	>2 doses	Total
<1	52	0	0	52
1–5	63	14	1	78
6–9	17	2	3	22
10–14	18	6	9	33
15–19	9	3	6	18
>20	77	4	3	84
Total	236	29	22	287


Most (71%, n = 204) case-patients were hospitalized. The most common signs and symptoms were maculopapular rash (100%), fever (100%), cough (75%), coryza (65.5%), and conjunctivitis (40%). The median duration of rash was 4 days. The most frequently reported complications were pneumonia (5%) and diarrhea (1%).

The index case-patient was a 5-year-old boy who had received 1 dose of the MMR vaccine and started having symptoms on February 4, 2023, after traveling through several countries in Europe. He exposed a nurse working in a pediatric clinic, initiating a transmission chain that lasted 13 generations and involved 177 cases (Table 2). Of the 287 total cases, 183 were linked to transmission chains, but 104 could not be linked. Among the 183 cases with a known exposure location, 129 (70%) were exposed in healthcare settings and 54 (30%) in community settings. Of the 104 unlinked cases, 66 had an unknown source of infection, and 38 were classified as imported, having been infected abroad.

**Table 2:** Characteristics of cases and transmission chains during measles outbreak driven by nosocomial transmission, Armenia, February–July 2023

Characteristic	Transmission chain			Not linked*
	1	2	3	
No. cases	177	4	2	104
No. in healthcare facilities	125	2	2	–
No. in community settings	52	2	0	–
No. generations	13	2	1	–
No. cases among healthcare workers	9	1	0	5
Vaccination status				
Not vaccinated	142	4	2	87
1 dose	21	0	0	9
2 doses	14	0	0	8

*Includes cases with unknown infection source and imported cases.

Unvaccinated or undervaccinated HCWs and nosocomial transmission contributed to this prolonged measles outbreak in Armenia. In addition, the high percentage of unvaccinated children highlights gaps in the childhood immunization program. Although Armenia achieved measles control nearing elimination during 2007–2022, national immunization coverage surveillance failed to identify inadequately immunized groups, which enabled sustained measles transmission for >5 months during 2023.

The most affected group was unvaccinated children <4 years of age. High national vaccination coverage did not ensure transmission control because immunity gaps persisted at subregional and community levels. Infants <1 year of age who had not yet reached the recommended age for immunization (n = 53) were mostly infected in healthcare facilities. As part of the outbreak response, children 6–9 months of age who were identified as close contacts of measles cases were offered vaccination. Cases among infants who are not yet eligible for vaccination pose a substantial challenge to achieving measles eradication (*2*,*3*). 

We implemented immediate control measures, including offering MMR vaccination to all inadequately immunized close contacts of confirmed cases (*6*). A total of 33,385 persons were immunized, including 6,693 HCWs. In addition, NCDC launched risk communication and awareness campaigns directed to the public and to persons traveling abroad. In addition, we recommended revising the national strategic plan for measles elimination to include mandatory immunization for front-line HCWs (*7*) and to strengthen monitoring of vaccination uptake within specific community subgroups.

## Conclusions

Our investigation revealed gaps in measles surveillance and infection prevention and control in Armenia. Limited resources prevented routine PCR testing and genotyping, and many children were not referred for testing because of parental refusal. In addition, some HCWs failed to adhere to isolation and infection control protocols. Continued transmission after August 2023, primarily occurring outside healthcare settings, highlighted the lack of effective measles control, driven by persistence of underimmunized groups and emergence of displaced populations after the Armenia–Azerbaijan conflict in September 2023. 

In summary, during this large measles outbreak, most case-patients were exposed in healthcare facilities, and unvaccinated HCWs played a key role in the spread of infection, especially in the first phase of the outbreak. To minimize the risk for nosocomial transmission, HCWs should be made aware of measles and the critical role of vaccination in measles prevention. 
